# Bazex Syndrome (Acrokeratosis Paraneoplastica): A Narrative Review of Pathogenesis, Clinical Manifestations, and Therapeutic Approaches

**DOI:** 10.7759/cureus.45368

**Published:** 2023-09-16

**Authors:** Muhammad Hamza Shah, Carlo Ferrazzano, Anhukrisha Karthikeyan, Hamad Hejazi, Anushri Bhattacharya, Wireko Andrew Awuah, Arda Isik

**Affiliations:** 1 Centre for Anatomy, Deanery of Biomedical Sciences, The University of Edinburgh, Edinburgh, GBR; 2 School of Medicine, Dentistry and Biomedical Sciences, Queen's University Belfast, Belfast, GBR; 3 Faculty of Medicine, Sumy State University, Sumy, UKR; 4 Surgery, University of Pittsburgh Medical Center, Pittsburgh, USA

**Keywords:** clinical manifestations, pathogenesis, oncology, paraneoplastic dermatosis, acrokeratosis paraneoplastica

## Abstract

Acrokeratosis paraneoplastica, also known as Bazex syndrome, is a relatively understudied yet clinically important dermatological condition that is inextricably associated with squamous cell carcinoma, predominantly of the upper respiratory and gastrointestinal tracts. Manifesting as unique psoriasis-like cutaneous lesions, Bazex syndrome functions as an early warning signal for underlying malignancies, underscoring the urgent need for its timely diagnosis and intervention. Notwithstanding its clinical relevance, the molecular and cellular mechanisms underpinning its pathogenesis are not fully understood. To address these gaps, this comprehensive literature review undertook a meticulous search across reputable databases such as EMBASE, MEDLINE/PubMed, and Web of Science. Our analysis ventures into multiple putative pathogenic pathways, including shifts in Th2 immune responses, aberrant secretion of growth factors, and immunological reactions to tumor-specific antigens. We also detail the clinical phenotypes of Bazex syndrome and their chronological linkage with the corresponding malignancies. Finally, the review evaluates the therapeutic effectiveness of various approaches, including but not limited to targeted cancer treatments, PUVA therapy, and topical corticosteroids. This synthesis aims to arm healthcare providers with a nuanced understanding of Bazex syndrome, facilitating more accurate diagnosis and tailored treatment paradigms.

## Introduction and background

Acrokeratosis paraneoplastica (APB) is an uncommon dermatological disorder classified as an obligate paraneoplastic dermatosis. Named after Bazex, who made a groundbreaking discovery in 1965, the syndrome forged a clear link between psoriasis-like skin lesions and squamous cell carcinoma (SCC) originating from the piriform fossa [1]. Characterized by painful, reddish patches and plaques exhibiting hyperkeratosis and scaling, APB primarily affects the palms, soles, fingers, and toes, with accompanying nail changes and additional skin manifestations, further complicating the diagnostic and management landscape [2]. The atypical distribution of psoriasis-like lesions, extending beyond the extremities to involve the cheeks, nose, and ears, marks APB with distinctiveness amidst other dermatological conditions.

Notably, the intrigue surrounding Bazex syndrome is heightened by its close association with specific types of cancers, prominently SCC arising from the upper respiratory and upper digestive tracts, such as the larynx, pharynx, and esophagus [3]. This inherent correlation transforms APB into a critical marker for underlying malignancies, necessitating early recognition to ensure timely and effective intervention and treatment. Meanwhile, the precise pathogenesis of APB remains somewhat elusive, albeit theories propose that cytokines or hormones produced by the associated tumor may trigger the observed skin abnormalities [4]. Deciphering the interplay between the dermatological manifestations and the underlying malignancies holds promise in unraveling the pathophysiological mechanisms driving this paraneoplastic syndrome.

Given the rarity and diagnostic challenges posed by APB, this article endeavors to delve deeper into its clinical presentation, pathogenesis, and the fundamental link to cancer development. Therefore, a comprehensive understanding of this syndrome assumes paramount importance for healthcare professionals in enabling early detection and implementing a tailored management strategy.

## Review

Methodology 

This literature review was undertaken from 15th July 2023 to 28th July 2023, employing a methodologically sound search strategy to ensure a comprehensive and up-to-date analysis. The primary databases, including EMBASE, MEDLINE/PubMed, and Web of Science, were deliberately selected for their extensive coverage of scholarly literature in the fields of dermatology and oncology.

To ensure adequate analysis of Acrokeratosis paraneoplastica, a set of critical keywords were curated. These encompassed fundamental aspects of the syndrome using keywords like "bazex syndrome," "paraneoplastic dermatosis" and "acrokeratosis paraneoplastica." Additionally, the PubMed search string was tailored to include terms related to diagnosis, prognosis, and management. Furthermore, in an endeavor to leave no valuable insight unexplored, an additional manual search was conducted by examining the reference lists of retrieved papers. This supplementary effort was integral to identifying any potentially significant publications that might have eluded the initial electronic search. Additionally, to ensure coherence and accessibility, a strict criterion was adhered to, considering only articles written in the English language for inclusion in this review. 

**Figure 1:**
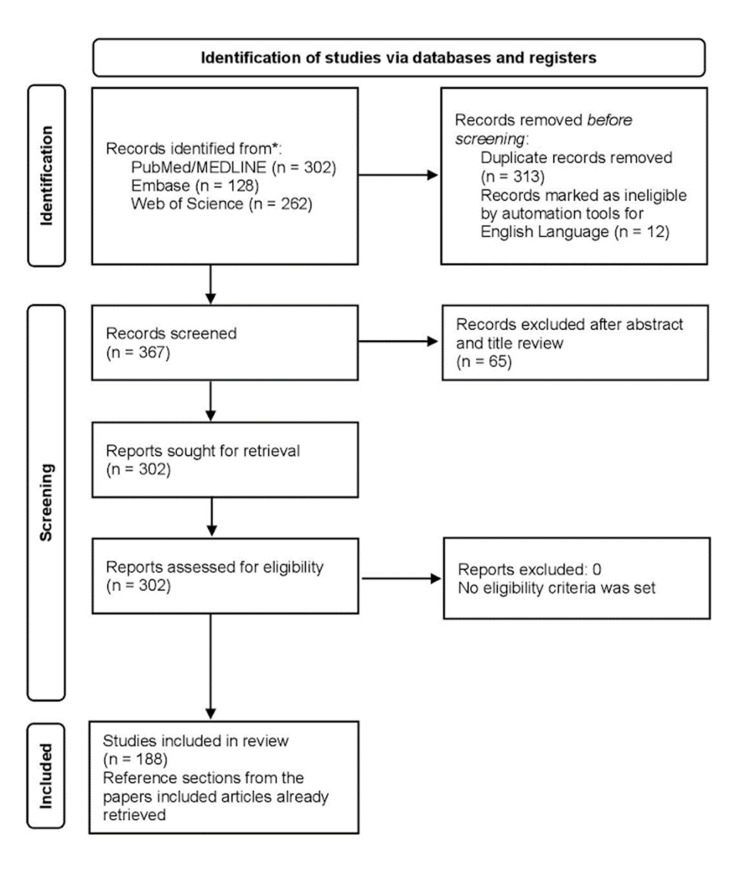
Modified PRISMA diagram showcasing the research strategy PRISMA: Preferred Reporting Items for Systematic Reviews and Meta-Analyses

Subsequently, the screening process was implemented to assess the relevance and quality of each identified paper. The methodological rigor of this literature review aligns with the guidelines set forth by the SANRA framework, upholding standards of research methodology and reporting. A summary of the methodology is illustrated in Figure 1. 

Pathogenesis

The pathogenesis of Bazex syndrome, colloquially known as acrokeratosis paraneoplastica, remains uncertain for the most part. Despite the lingering ambiguities, several theoretical frameworks closely tie the syndrome's onset to underlying malignancies, particularly squamous cell carcinomas (SCCs). Delving into these frameworks, three primary pathways emerge:

Pathway 1

**Figure 2:**
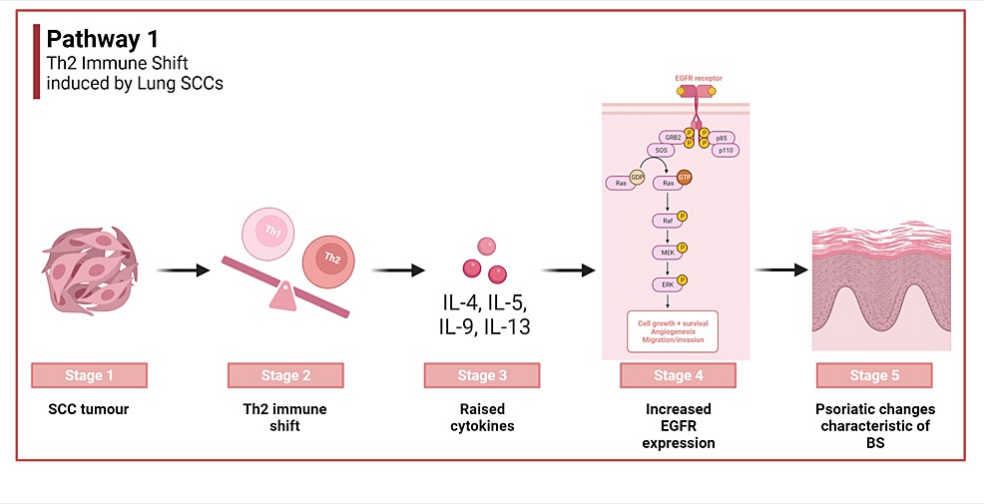
Th2 immune shift induced by lung squamous cell carcinoma SCC: Squamous cell carcinoma, Th-2: T helper type 2, IL-4: interleukin-4, IL-5: interleukin-5, IL-9: interleukin-9, IL-13: interleukin-13, EGFR: epidermal growth factor receptor, BS: Bazex syndrome

Th2 immune shift induced by lung SCCs:Lung SCCs may elicit a distinct immune response, ushering in a Th2 immune shift. This altered immune paradigm manifests through heightened levels of serum markers like IgE, thymus and activation-regulated chemokine (TARC), and eosinophils [5]. Within this scenario, Th2 cells liberate a spectrum of cytokines, notably interleukins 4, 5, and 13, thereby fostering allergic hypersensitivity reactions. The role of interleukin-10, another product of Th2 cells, cannot be understated, given its anti-inflammatory prowess [6]. This overarching Th2-centric response seemingly augments epidermal growth factor receptor (EGFR) expression in keratinocytes, which have suffered lesions. As EGFR levels surge, there's a propensity for acral psoriasiform lesions and other cutaneous manifestations emblematic of Bazex syndrome to develop. Interestingly, excising the neoplastic trigger, often via lobectomy, recalibrates the immune landscape, ameliorating the skin symptoms [5]. Pathway 1 is depicted in Figure 2. 

Pathway 2

Growth factor production by SCC tumors: In juxtaposition to the immunological focus, an alternative hypothesis postulates that during the proliferative phase of SCC tumors, there is an orchestrated secretion of distinct growth factors. Predominantly, this includes Transforming Growth Factor-alpha (TGF-α), Epidermal Growth Factor (EGF), and Insulin-like Growth Factor-1 (IGF-1). Acknowledged for their integral roles in modulating cellular proliferation, these growth factors are conjectured to stimulate hyperproliferation of epidermal and epithelial cells, thereby significantly influencing the dermatological manifestations observed in Bazex syndrome [7].

Pathway 3

**Figure 3:**
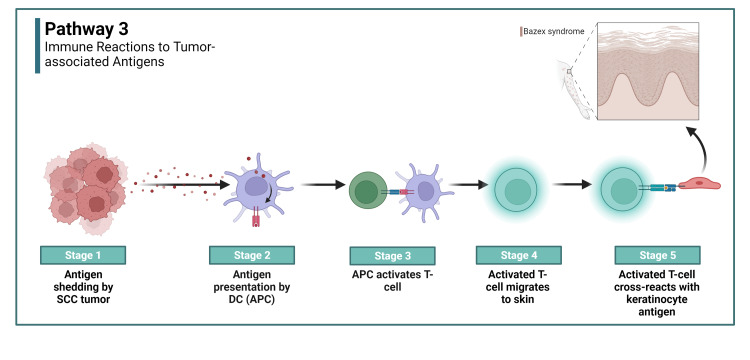
Immune reactions to tumor-associated antigens SCC: Squamous cell carcinoma, DC: dendritic cell, APC: antigen presenting cell

Immune reactions to tumor-associated antigens: Adding another layer to the intricate pathogenesis is the potential role of tumor-associated antigens, specifically those emanating from SCCs. When these antigens infiltrate the immune system, the body mounts a defensive antibody response. The ensuing antibodies, crafted to neutralize these tumor antigens, might inadvertently cross-react with antigens present on keratinocytes or the basement membrane [8,9]. Alternatively, the body might mount a T-cell-mediated immune response against the tumor antigens, but the cytotoxic T-cells may cross-react with keratinocyte antigens. Such unintended interactions pave the way for autoimmune reactions, leading to potential damage, particularly targeting the basement membrane [9]. Pathway 3 is depicted in Figure 3. 

Clinical manifestations

The clinical manifestations often include plaques that vary in color from reddish to bluish-purple with noticeable scaling [10]. This scaling superficially resembles psoriasis; however, the lesions in Bazex syndrome may occur in regions uncommonly associated with psoriasis vulgaris [11]. The defining clinical characteristic of this syndrome lies in the involvement of the external helices of the ears and the nose tip [12]. Among the most frequently affected sites are the ears, nails, nose, fingers, palms, hands, soles, and feet. On a less common note, the knees and elbows can exhibit lesions in approximately 20% of patients, usually later in the disease's course [12-16].

Variations 

Despite the general consistency in the symptomatology of Bazex syndrome across different cases, one noticeable disparity lies in the temporal relationship between skin manifestations and the appearance of the underlying malignancy symptoms [7,10,11,13]. The skin changes precede cancer symptoms by at least six months in a majority of cases, but this pattern does not apply universally. Approximately, 30% of reported cases document skin changes manifesting post-detection or symptomatic onset of the malignancy [13,14,16].

Phased Clinical Manifestations

Stage 1: Initial symptomatology includes symmetrical invasion of the ear helices, nose, fingers, and toes. Early plaques are flat and poorly demarcated, sometimes exhibiting crusts and scaling, typically devoid of associated symptoms. Pruritus, however, is a commonly reported concern [11,14].

Stage 2: As the underlying neoplasm advances, potentially leading to localized or metastatic spread, a more extensive skin outbreak ensues. Characteristic red to purple scaly or crusted plaques emerge on the cheeks, with scaling developing on the palms and soles, excluding the central areas. Fissuring, primarily on the feet, and nail pathology, manifesting as yellowing, thickening, onycholysis, and both horizontal and vertical ridging, may result in pain and disability [13,15,16].

Stage 3: This final stage coincides with the untreated or treatment-resistant carcinoma. Pre-existing signs and symptoms persist, while papulosquamous lesions begin to present on the trunk, elbows, knees, and dorsal hands and feet. In some instances, vesicles and bullae may emerge, mainly on the fingers, hands, and feet. Nail changes can vary significantly, ranging from typical thickening to atrophy and even complete loss [12,15].

Malignancies 

To elucidate the relationship between Bazex Syndrome and various types of cancer, Table 1 categorizes each published case by the type of cancer associated and the number of available case reports, with the corresponding author and publication year. Among this list, lung cancer appears most frequently, followed by other types such as oral cancer and head and neck cancer.

**Table 1:** Association between Bazex syndrome and types of cancers based on published case reports

Serial Number	Cancer Associated	Number of case reports	Author and Publication Year
1	Lung Cancer	11	Ikeda et al., 2022 [17]; Mititelu et al., 2019 [16]; Aoshima et al., 2019 [18]; Amano et al., 2016 [5]; Zhao et al., 2016 [19]; George et al., 2017 [20]; Fleming et al., 2014 [21]; Crucitti et al., 2009 [22]; Sharma et al., 2006 [11]; Valdivielso et al., 2005 [23]
2	Hepatocellular Cancer	2	Holzgruber et al., 2022 [10]; Matsui et al., 2016 [24]
3	Acute Myeloid Leukemia	1	Oka et al., 2023 [25]
4	Lip Cancer	1	Gaurav et al., 2022 [26]
5	Oral Cancer	7	Dabas et al., 2018 [27]
6	Pancreatic Cancer	4	Horton et al., 2020 [28]; Narasimha et al., 2020 [29]; Iwanami et al., 2017 [30]; Halpern et al., 1995 [31]
7	Head and Neck Cancer	7	Eckstein et al., 2020 [8]; Duran-Vian et al., 2020 [32]; Adelman et al., 2018 [33]; Shikino et al., 2017 [34]; Driessen et al., 2013 [35]; Koh et al., 2012 [36]; Asokan et al., 1996 [37]
8	Oesophageal Cancer	7	Graves et al., 2014 [38]; Humphrey et al., 2015 [39]; Rodrigues et al., 2013 [40]; Louvel et al., 2008 [41]; Medenica et al., 2008 [42]; Poligone et al., 2007 [43]; Viteri et al., 2005 [44]
9	Papillary Thyroid Cancer	1	Macca et al., 2022 [45]
10	Hodgkin’s Lymphoma	2	Nguyen et al., 2019 [46]; Lucker et al., 1995 [47]
11	Peripheral T-cell Lymphoma	1	McClatchey et al., 2018 [48]
12	Prostate Cancer	1	Figueroa-Silva et al., 2017 [49]
13	Follicular Lymphoma	1	Conde-Montero et al., 2017 [50]
14	Cervical Cancer	3	Squires et al., 2016 [51]; Wareing et al., 1996 [52]; Richard et al., 1987 [53]
15	Gastric Cancer	2	Kofler et al., 2015 [54]; Robert et al., 2014 [55]
16	Colorectal Cancer	3	Baek et al., 2008 [56]; Rao et al., 2004 [57]; Hsu et al., 2000 [13]
17	Basal Cell Cancer	1	Griffin et al., 2007 [58]
18	Breast Cancer	2	Taher et al., 2007 [59]; Akhyani et al., 2004 [60]
19	Tonsillar Adenocarcinoma	1	Ali et al., 2004 [61]

Therapeutic approaches

Management of Bazex syndrome is complex. There are several factors at play that influence the resolution of the clinical signs and symptoms of the pathology. Often, patients respond differently to the variety of treatments encountered in the literature. 

Treatment of Underlying Malignancy

Given Bazex syndrome is a paraneoplastic manifestation, the vast majority of cases reported refer to a complete or partially complete resolution of symptoms with treatment of the underlying malignancy [31]. As mentioned earlier, Bazex syndrome evolves in three stages that are complementary to the growth of the tumor. Therefore, early recognition of the syndrome in its primary stage can aid the prompt diagnosis and management of the underlying cancer [62]. The most commonly associated malignancies involve the oral and respiratory tracts. These tumors are often treated with a combination of chemotherapy, radiotherapy and surgical resection [11]. For example, a 53-year-old man with squamous cell carcinoma of the tonsil with lymph node involvement was treated with radiotherapy leading to a near complete resolution of the of the skin lesions [12]. Another patient with adenocarcinoma of the lung was treated with Gefitinib, a receptor tyrosine kinase inhibitor, which reduced tumor and cutaneous lesion size [19]. 

Additionally, due to the intrinsic nature of Bazex syndrome resembling psoriasiform or eczematous lesions, often treatments for psoriasis and eczema, both topical and systemic, are used but are mostly ineffective [40]. Even though some improvement in the cutaneous lesions has been reported with these treatments, clearance most often fails as long as there is persistence of the underlying tumor [13,63]. Bolognia et. al. suggested this is the case for 91% of the patients (64/70) encountered in the literature [64]. In some cases, patients also present with nail changes such as dryness, increased thickness and other onychodystrophy changes. While cutaneous manifestations tend to resolve with the treatment of the underlying malignancy, some reports suggest nail changes can persist and take longer to resolve [65,66]. Furthermore, lesions tend to reappear as a result of neoplastic recurrence [39,62,67,68]. Therefore, it is important to observe the full clinical picture as reappearance of the lesions may signify tumor recurrence or development of metastatic disease. Some cases report a usual persistence of acrokeratosis after treatment of underlying neoplasm. For instance, a 40-year-old man with an oropharyngeal carcinoma achieved complete tumor remission without resolution of psoriasiform lesions [69]. Another patient with an unusual presentation involving Bazex syndrome and two different neoplasms of the prostate gland and an undifferentiated carcinoma of the submandibular gland showed minimal improvement in cutaneous symptoms and died four months later as a result of metastatic dissemination [49]. Rarely, the cutaneous eruptions spontaneously resolve without any treatment involving the skin itself or the underlying malignancy. This has only been reported in three cases so far [63]. 

Oral psoralen-UVA phototherapy: Oral psoralen-UVA phototherapy, commonly known as PUVA therapy, has emerged as a promising treatment for certain skin conditions, particularly in patients with Bazex syndrome. Recent studies, such as the one conducted by Gill and colleagues, have underscored the potential efficacy of this method [70]. Their findings suggest that patients experience notable improvement, with some achieving complete clearance of cutaneous lesions after undergoing 18 cycles of PUVA therapy over a span of just three weeks. This therapy involves the administration of oral psoralen, followed by UV-A phototherapy. Additionally, there have been individual cases where patients have experienced significant symptomatic relief after PUVA therapy. In one notable instance, a female patient with pronounced skin lesions underwent treatment with psoralen ultraviolet A therapy, leading to the complete resolution of the cutaneous changes [71]. 

Steroids: Steroids, both topical and systemic, have been reported as potential therapeutic agents for Bazex syndrome. Topical treatments include corticosteroids such as clobetasol 0.05% and betamethasone 0.01%. Other topical ointments reported include salicylic acid 10% in vaseline, itraconazole, isosorbide dinitrate, fluconazole, cephalexin, keratolytics, neomycin, nystatin, zinc ointment, antibiotics, and emollients [2]. As for oral steroids, the recommended starting dose is prednisolone at 0.5 mg/kg/day, with a suggested weaning over a month or starting with an initial dose of 60 mg/day [2,72]. Some research also indicates the effectiveness of oral dexamethasone at 10 mg/day, which has led to favorable outcomes in the treatment of cutaneous lesions [35]. While many studies have highlighted good-to-moderate response rates from topical steroids, a few authors question the tangible benefits of their use [19,66]. 

Etretinate and acitretin: Oral retinoids have been identified as yet another avenue for treating cutaneous manifestations in Bazex syndrome, especially when primary tumor treatments prove ineffective. Notably, etretinate, an aromatic retinoid, demonstrated improvement in these skin lesions [26]. Acitretin has also been effective, providing relief to at least two patients in a documented case series [8]. Interestingly, one of these patients experienced a recurrence and even progression of cutaneous symptoms and burning sensations after discontinuing acitretin treatment, despite being cancer-free. Reinitiating the acitretin therapy led to a subsequent improvement in the symptoms. These cases suggest that oral retinoids like etretinate and acitretin not only can be effective in reducing hyperkeratosis but may also be crucial in maintaining symptom control even after successful cancer treatment.

Discussion

Bazex syndrome, also known as Acrokeratosis paraneoplastica, is a paraneoplastic cutaneous disorder commonly associated with SCC of the upper aerodigestive tract. This review outlines the underlying mechanisms responsible for the disease's onset, including the role of specific immune pathways and growth factors. It also catalogs the various dermatological symptoms, providing a framework for diagnosis. Lastly, the paper evaluates existing treatment methods and introduces new approaches for managing the condition. 

As noted by our review, APB presents quite uniformly across cases, often involving acral sites, the ears, and nose tip [2,12]. However, a noteworthy variance lies in the temporal onset of these symptoms relative to the appearance of cancer symptoms. While in most cases, the skin changes precede the cancer manifestations, this pattern does not universally hold [13,15]. This discrepancy suggests that the disease's clinical course may not be strictly linear and that several factors, potentially including the type and stage of the underlying cancer, may influence the timing and severity of cutaneous symptoms. Furthermore, the available literature makes a strong case for a three-staged development pattern for APB, progressing from initial invasion of the acral sites and ears to more widespread skin manifestations as the underlying malignancy progresses [14,16]. This pattern suggests that a dynamic relationship exists between the cutaneous symptoms and the underlying malignancy, further underlining the critical importance of treating the root cancer for managing APB symptoms effectively. 

Treatment modalities for the disease range from surgical interventions to chemotherapy and radiotherapy for the underlying cancer. Importantly, our review emphasizes that management of the underlying malignancy often results in the resolution of APB symptoms, reinforcing the syndrome’s role as a marker for malignancy [30]. Traditional treatments for dermatological conditions like psoriasis and eczema generally show limited efficacy in managing APB symptoms [40]. This could be attributed to the syndrome's paraneoplastic nature, and persistence of the underlying tumor often corresponds with unresponsiveness to these treatments. In certain instances, therapies like PUVA have shown promise [70,71], but the primary focus remains on treating the underlying malignancy. Steroids, both topical and systemic, present another avenue for symptom management but with mixed results [2,19,33].

In summary, the importance of a multifaceted approach to diagnosing and managing APB, taking into account both the cutaneous symptoms and the underlying malignancy, is evident from the reviewed literature. Future research should focus on a more comprehensive understanding of the complex pathogenesis, which could potentially open new avenues for targeted therapies and better management strategies.

Limitations

While this study aims to provide a comprehensive overview of Bazex syndrome, certain constraints could affect the interpretation of its conclusions. The current literature is notably limited, often based on case reports or small cohort studies, which may not offer a comprehensive view and could potentially introduce bias. The generalizability of these findings to a broader population is, therefore, a subject for further investigation. Similarly, the underlying mechanisms posited for the syndrome's etiology are still in the realm of speculation, given the relatively unexplored territory of molecular and cellular research in this specific condition. The absence of universally accepted diagnostic criteria also introduces a layer of complexity, potentially leading to variations in case identification and evaluation of treatment efficacy. Moreover, the therapeutic approaches suggested in this study are not backed by extensive evidence, particularly from randomized controlled trials, making them tentative recommendations at best.

## Conclusions

APB serves as a dermatological harbinger for specific types of malignancies, chiefly SCCs. It presents a diagnostic challenge but offers a unique opportunity for early cancer detection. While our understanding of its pathogenesis is growing, concrete evidence remains elusive, necessitating further in-depth investigations. As for treatment, a multidisciplinary approach targeting both the dermatological manifestations and underlying malignancy seems most effective. As we continue to elucidate the complex mechanisms behind APB, there is hope that future research will offer more targeted and effective therapeutic strategies.
